# Cerebral Hemodynamic Patterns in People with Subjective Cognitive Decline: A Systematic Review Based on Functional Near-Infrared Spectroscopy

**DOI:** 10.3390/brainsci16101130

**Published:** 2026-10-20

**Authors:** Carolina del Pilar Pebe-Muñoz, Patricia Alzola, Emiliano Díez-Villoria, Israel Contador

**Affiliations:** 1 Department of Basic Psychology, Psychobiology and Methodology of Behavioral Sciences, Faculty of Psychology, University of Salamanca, 37005 Salamanca, Spain; 2 Instituto Universitario de Integración en la Comunidad (INICO), University of Salamanca, 37005 Salamanca, Spain; 3 Department of Neurobiology, Care Sciences and Society, Aging Research Center, Karolinska Institutet and Stockholm University, 17165 Stockholm, Sweden

**Keywords:** subjective cognitive decline, memory complaints, fNIRS, cognition, neuropsychology, neuroimaging

## Abstract

**Highlights:**

**What are the main findings?**

- Subjective cognitive decline (SCD) has been associated with heterogeneous brain hemodynamic responses using functional near-infrared spectroscopy (fNIRS). The limited evidence and methodological variability make it challenging to identify a specific hemodynamic pattern for SCD.

**What are the implications of the main findings?**

- Understanding the brain hemodynamic responses underlying SCD may be essential for the early detection and prevention of neurocognitive disorders.
- Future research requires longitudinal designs, standardized diagnostic and fNIRS procedures, and challenging tasks to better differentiate hemodynamic responses among cognitively healthy, SCD, and cognitively impaired groups.

**Abstract:**

**Background/Objectives**: Subjective cognitive decline (SCD) has been proposed as an early symptomatic manifestation of dementia/Alzheimer’s disease. However, its associated brain patterns are not yet fully characterized. In recent years, functional near-infrared spectroscopy (fNIRS) has emerged as a cost-effective technique to detect hemodynamic changes associated with cognitive decline. Therefore, fNIRS may be a valuable tool to investigate SCD neural underpinnings. This systematic review synthesizes current fNIRS evidence regarding the hemodynamic correlates of SCD during both resting-state and cognitive task conditions. **Methods**: Following PRISMA guidelines, we searched Scopus, PubMed, and Web of Science databases for studies combining keywords (‘subjective cognitive decline’, ‘cognitive complaints’, ‘memory complaints’, ‘fNIRS’, and ‘near-infrared spectroscopy’) with Boolean operators. **Results**: We identified 181 records, of which 10 studies fulfilled the inclusion and exclusion criteria. All studies were cross-sectional. Furthermore, 33% lacked a healthy control group, 56% lacked a standardized SCD identification protocol, 55% used small or imbalanced samples, and 66% did not clearly differentiate prefrontal subregions. In both resting-state and task conditions, SCD individuals showed heterogeneous hemodynamic responses in prefrontal areas. The severity of SCD and the difficulty of the tasks may be useful moderators of these associations. **Conclusions**: The limited evidence and methodological differences prevent identifying a specific hemodynamic pattern for SCD. Adopting longitudinal, well-powered, and well-controlled designs will be essential to evaluate fNIRS-derived hemodynamic responses in SCD.

## 1. Introduction

Dementia is one of the leading causes of disability and dependency in older adults [1]. Its prevalence is expected to increase. Specifically, European Union countries estimate a 58% rise in dementia cases between 2025 and 2050 [2]. Consequently, dementia is a major public health priority that requires effective strategies to meet the needs of patients and their caregivers [3]. Among dementia types, Alzheimer’s disease is the most common type, accounting for 60% to 70% of cases [1]. This neurodegenerative disease is characterized by progressive deterioration in episodic memory, executive functions, language, praxis, and visual perception, leading to loss of functionality [4,5]. Identifying the early symptoms that predict cognitive decline progression enables the development of tailored prevention methods [6].

Given the gradual nature of cognitive decline in Alzheimer’s disease, a clinical continuum has been proposed [7]. This continuum includes (i) subjective cognitive decline (SCD); (ii) mild cognitive impairment (MCI), characterized by significant cognitive deterioration with preserved independence; and (iii) dementia, which affects multiple cognitive domains and compromises an individual’s functionality and autonomy. Within this continuum, the Subjective Cognitive Decline Initiative (SCD-I) research group defines SCD as a self-perceived progressive cognitive decline without a specific cause, where performance on standardized neuropsychological tests falls within normal ranges [8,9].

Individuals with SCD have a twofold risk of progression to cognitive impairment and dementia [10,11]. This has increased research interest in the topic [12]. However, some evidence shows similar dementia progression rates between individuals with SCD (≈7%) and without SCD (≈6%) [13]. Thus, subjective cognitive complaints are not always associated with Alzheimer’s disease development [14]. Moreover, identifying SCD remains challenging because it is often considered a normal symptom of aging, cognitive performance remains within normal parameters, and no gold-standard diagnostic reference exists [9]. In the scientific literature, several factors have been associated with SCD, including psychological distress (e.g., anxiety), personality traits (e.g., neuroticism), vascular risk factors, or endocrine disorders (e.g., hypothyroidism) [9,14]. Low cognitive reserve, physical inactivity, and sleep problems [15,16,17] also make SCD characterization particularly challenging.

Although individuals with SCD maintain normal cognitive scores, evidence suggests subtle neurophysiological and structural changes in the brain supporting memory, language, and executive functions [18]. At the anatomical level (e.g., magnetic resonance imaging, MRI), reduced cortical thickness and lower gray matter volume have been reported in the temporal regions of participants with SCD, particularly in the hippocampus [19]. Studies addressing SCD at the functional level (e.g., functional MRI, fMRI) have reported regional hyperactivation in prefrontal, parietal, temporal, and limbic regions, which may reflect compensatory mechanisms [19,20,21,22,23,24]. Additionally, altered default mode network (DMN) deactivation has also been reported in SCD, which may indicate reduced cognitive resource allocation in this population [25].

Positron emission tomography (PET), single-photon emission computed tomography (SPECT), and fMRI have proven useful in the early detection of neurodegenerative diseases [26,27]. However, these brain imaging methods are costly, invasive, and require specialized staff [26,27]. In this context, functional near-infrared spectroscopy (fNIRS) emerges as a promising alternative, providing practical advantages such as cost-effectiveness, silent operation, motion tolerance, portability, and non-invasiveness [28]. This technique uses near-infrared light, which penetrates tissue and is absorbed by oxygenated (HbO) and deoxygenated (HbR) hemoglobin [29]. fNIRS relies on neurovascular coupling, a mechanism by which cortical activity leads to increased cerebral blood flow [30]. Accordingly, an increase in HbO and a decrease in HbR are observed in active regions [31], reflecting neuronal functionality [32].

The fNIRS technique enables the study of hemodynamic responses during the resting-state [28] and during cognitive task performance [27,33]. In various studies, fNIRS has differentiated cortical oxygenation patterns across the Alzheimer’s disease continuum (i.e., healthy older adults, SCD, MCI, and Alzheimer’s disease) [31]. However, no study has synthesized the available evidence in individuals with SCD. Therefore, the objective of this systematic review is to investigate fNIRS-derived hemodynamic correlates in older adults with SCD. Specifically, we aim to identify cortical activation patterns in SCD both at the resting-state and while performing cognitive tasks.

## 2. Materials and Methods

### 2.1. Study Design

This study was conducted in accordance with the PRISMA (Preferred Reporting Items for Systematic Reviews and Meta-Analyses) guidelines [34]. This systematic review has been registered in the Open Science Framework (OSF) and can be accessed via the following link: https://doi.org/10.17605/OSF.IO/2VU7W.

### 2.2. Eligibility Criteria

Studies were selected based on predefined inclusion and exclusion criteria. The inclusion criteria were (i) empirical research articles published in English or Spanish in peer-reviewed, indexed journals; (ii) sample populations including individuals with SCD; and (iii) analysis of cortical activity using fNIRS. Conversely, exclusion criteria were (i) theoretical articles, book chapters, systematic reviews, meta-analyses, or grey literature (e.g., conference abstracts, preprints, dissertations); and (ii) samples with cognitive complaints associated with a pre-established medical condition (e.g., cancer, hypothyroidism, vitamin B12 deficiency, fibromyalgia), severe psychiatric disorders (e.g., schizophrenia, major depression, and bipolar disorder), neurological diseases (e.g., stroke, traumatic brain injury, multiple sclerosis), or substance intake (e.g., alcohol, drugs, antipsychotics). The review team applied language criteria based on its proficiency.

### 2.3. Search Strategy

The units of analysis included in this review are empirical articles published in Scopus, PubMed, and Web of Science. One author (C.d.P.P.-M) conducted the literature search in February 2026. The keywords used were “subjective cognitive decline,” “cognitive complaints,” “memory complaints,” “fNIRS,” and “near-infrared spectroscopy” linked by the Boolean operators (“AND”/“OR”). Consequently, the search equation was (“subjective cognitive decline” OR “cognitive complaints” OR “memory complaints”) AND (“fNIRS” OR “near-infrared spectroscopy”). By default, searches targeted title, abstract, and keywords fields, adapted to the database-specific syntax (i.e., [TITLE-ABS-KEY] for Scopus, [Title/Abstract] for PubMed, and [TS] for Web of Science). This keyword combination was executed independently in each database without initial automated filters to maximize article retrieval. Study identification was determined by two independent reviewers (C.d.P.P-M. and I.C.).

Reference lists of all included studies were manually searched to identify additional eligible records, adding one study through this method. Neither forward nor backward citation chaining was used. When necessary, corresponding authors were contacted by email to request full-text manuscripts. All retrieved studies were exported to the reference management software Zotero (version 9.0.6) for duplicate removal and citation management.

### 2.4. Data Synthesis and Quality Assessment

Following full-text screening, data were extracted on sample characteristics, SCD criteria, covariates, experimental condition (resting-state or cognitive tasks), hemodynamic parameters, and main cognitive and hemodynamic outcomes. Statistical outcomes (e.g., effect sizes or test statistics) were extracted across studies, presenting ranges when necessary, to provide readers with a general view of quantitative results.

Study quality was determined by two independent reviewers (C.d.P.P-M. and I.C.) using the Oxford Centre for Evidence-Based Medicine [35] framework. Five levels were considered: (1) randomized clinical trial or systematic review/meta-analysis of several studies; (2) prospective cohort study; (3) retrospective cohort study, case–control study, or systematic review of several studies; (4) case series or cross-sectional studies; (5) single case study or expert opinions.

Risk of bias was evaluated using the Joanna Briggs Institute (JBI) Critical Appraisal Checklist for Analytical Cross-Sectional Studies [36]. This tool evaluates eight domains: (1) clear definition of sample inclusion criteria (e.g., systematic exclusion of medical, psychiatric, or neurological conditions); (2) use of objective, standardized criteria for condition measurement (e.g., SCD diagnosis based on formal consensus framework criteria or validated quantitative scales); (3) valid and reliable measurement of exposure (i.e., standardized execution of resting-state or task-based experimental paradigms); (4) valid and reliable measurement of outcomes (e.g., fNIRS technical quality, specification of regions of interest, and neuropsychological performance tracking); (5) identification of confounding factors (e.g., baseline sociodemographic or psychological variables); (6) strategies to deal with confounding factors (e.g., statistical control using ANCOVA/multivariable adjustment, a priori matching by design, or confirmed baseline homogeneity); (7) use of appropriate statistical analysis (e.g., suitable statistical tests for the data, adjustments for multiple channel comparisons, significant cohort sizes, and reports on effect sizes); and (8) detailed description of study subjects and recruitment setting description. All items are rated as Yes, No, Unclear, or Not Applicable. To align with the objectives of this systematic review, the overall appraisal for risk of bias was conducted by selecting the most relevant criteria based on JBI methodology. Four domains related to internal validity (i.e., Questions 2, 4, and 6) and statistical rigor (i.e., Question 7) were designated as critical. Studies were categorized as “Low risk” if all core critical domains were satisfied, “Moderate risk” if 1–2 critical domains presented unclear or unfulfilled criteria without compromising overall study integrity, and “High risk” if multiple critical domains were unfulfilled.

## 3. Results

**Figure 1:**
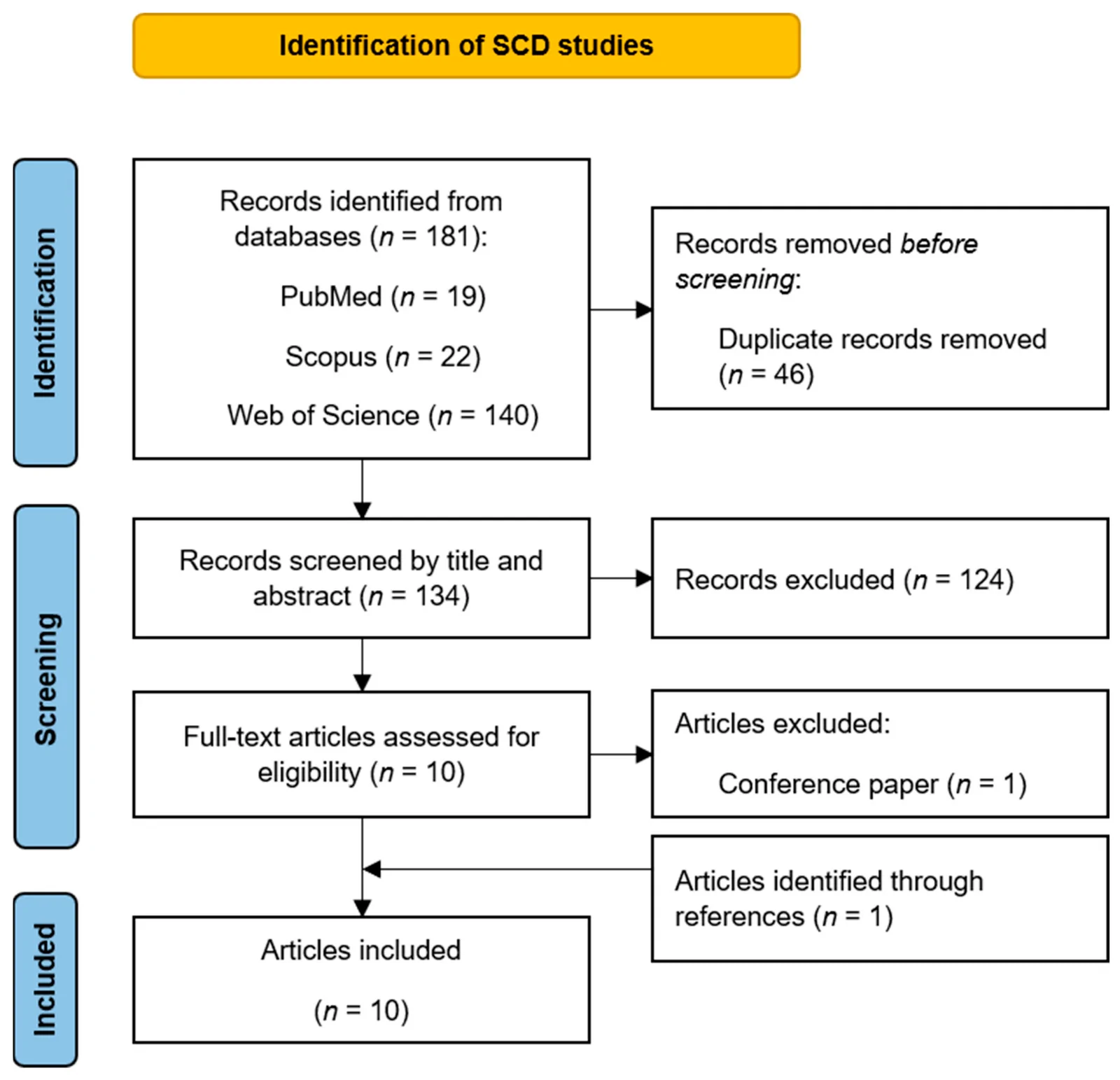
PRISMA flow diagram for article selection [34].

In this study, a total of 181 documents were identified. After deleting duplicates (*n* = 46) and screening titles and abstracts, ten studies fulfilled the criteria for this systematic review. Two of these studies used the same sample [37,38]. Thus, the results presented below correspond to nine independent samples. The screening process is shown in Figure 1.

Table 1 summarizes the main characteristics and key findings of the included studies. All investigations employed a cross-sectional design, classified as Level 4 of evidence. Regarding quality assessment, three studies (targeting two study samples) were categorized as “low risk”, four as “moderate risk”, and three as “high risk.” Table S1 (Supplementary Materials) details the risk-of-bias ratings for each study. Notably, all studies used continuous wave fNIRS over the prefrontal cortex to assess participants’ hemodynamic responses. In addition, a range of adjusted covariates was considered, including sociodemographic (e.g., age, sex, and education) and psychological factors (e.g., anxiety and depression). Table S2 also shows the cognitive performance results of each study. Finally, the PRISMA and PRISMA-S checklists can be found in Tables S3 and S4.

### 3.1. Hemodynamic Responses During Resting-State

Evidence from resting-state studies suggests that individuals with SCD exhibit increased prefrontal hemodynamic activity. One study examined functional connectivity (FC) in cognitively healthy, SCD, and MCI groups during a 5 min resting-state condition [37]. FC was assessed using HbO, HbR, and total hemoglobin time-series data to evaluate synchronization; that is, the temporal coordination of hemodynamic activity between prefrontal regions. Individuals with SCD showed greater HbR-based FC between the left dorsolateral prefrontal cortex and the right orbitofrontal cortex than cognitively healthy and MCI groups. Similarly, other authors evaluated FC in SCD and MCI groups during a 7 min resting-state [43]. The SCD group demonstrated stronger FC than the MCI group across most prefrontal channels, although reduced FC was also observed in a few prefrontal channels in individuals with SCD.

A recent study assessed FC and HbO concentration in cognitively healthy, SCD, MCI, and Alzheimer’s disease subgroups during a 5 min resting-state condition [46]. These authors observed higher HbO levels in the bilateral frontopolar cortex in both the SCD and MCI groups compared to the healthy control group. However, only SCD subjects showed higher HbO concentration in the left dorsolateral prefrontal cortex compared to healthy controls. The same study found that FC was stronger in the cognitively healthy group than in the SCD, MCI, and Alzheimer’s disease groups. FC was also greater in individuals with SCD than in those with Alzheimer’s disease. However, no significant differences were found between the SCD and MCI groups. Noteworthy, prefrontal hemodynamic responses were positively associated with Mini-Mental State Examination (MMSE) and Montreal Cognitive Assessment (MoCA) scores across the SCD, MCI, and Alzheimer’s disease groups.

### 3.2. Hemodynamic Responses During Cognitive Tasks

During cognitive task performance, several studies reported increased HbO concentrations and FC in the prefrontal cortex of participants with SCD. Further, the magnitude of these responses varied according to cognitive domain, task demands, and cognitive complaint severity.

#### 3.2.1. Global Cognition

Only one study evaluated global cognition during fNIRS recording [38]. This study assessed FC using HbO, HbR, and total hemoglobin time-series data in cognitively healthy, SCD, and MCI groups during the MMSE. Regarding HbO-based FC, the SCD group showed lower connectivity between the right dorsolateral prefrontal cortex and the right orbitofrontal cortex than cognitively healthy controls. In contrast, subjects with SCD showed greater connectivity between these areas than the MCI group. Regarding HbR-based FC, both the SCD and MCI groups demonstrated weaker connectivity between the left dorsolateral prefrontal cortex and the left ventrolateral prefrontal cortex than cognitively healthy controls. According to MMSE performance, people with SCD exhibited longer response times than cognitively healthy controls. However, they showed shorter response times and higher scores than the MCI group.

Overall, these findings suggest that distinct prefrontal FC patterns and cognitive performance using a global cognition task emerge across the different levels of cognitive functioning.

#### 3.2.2. Memory

Three studies incorporated a memory task [38,39,43]. One of them investigated brain activity by measuring HbO concentration during a visual memory span task in aSCD (mild, moderate, and severe) and MCI subtypes [39]. The authors observed lower HbO concentrations in the prefrontal cortex of individuals with severe aSCD than in those with mild aSCD and cognitively healthy controls. Under higher cognitive demands (i.e., a larger number of items to remember), the severe aSCD group exhibited poorer hemodynamic activation than the mild aSCD group. No significant group differences were detected in memory task performance. Nevertheless, the HbO concentration was positively associated with word-list learning scores across all aSCD levels and the cognitively healthy group, suggesting a relationship between memory performance and hemodynamic responses. Likewise, another study assessed FC using HbR time-series data in cognitively healthy, SCD, and MCI groups during a word-learning task [38]. Participants with SCD and MCI showed poorer FC between the left dorsolateral prefrontal cortex and the right medial prefrontal cortex than cognitively healthy controls. At the cognitive level, the SCD group performed worse than the cognitively healthy group but achieved higher memory scores than the MCI group. In contrast, other authors observed no significant differences in HbO concentrations between SCD and MCI groups during an image-recall task [43]. In this study, no statistically significant discrepancies were observed in accuracy or reaction times, although the MCI group tended to score lower than the SCD group.

In summary, compared to cognitively healthy controls, individuals with SCD exhibited lower prefrontal activity and reduced FC during memory tasks. Furthermore, greater memory complaint severity was associated with reduced prefrontal activation. However, these differences were not consistently observed across all studies.

#### 3.2.3. Executive Function

Six studies incorporated an executive function task [40,41,42,43,44,45]. One study examined activation in the left and right prefrontal cortex by measuring HbO concentration during n-back tasks in individuals with aSCD [44]. Participants were classified into high- and low-activation levels based on bilateral prefrontal activation. Amnestic SCD individuals with higher activation showed greater HbO concentrations during the 2-back condition than those with lower activation. Both groups (i.e., high and low activation) exhibited lower accuracy and longer response times in the 2-back task than in the 0-back task. However, aSCD individuals with higher activation achieved greater response accuracy compared to those with lower activation. Moreover, these authors analyzed whether prefrontal hemodynamic responses in the 2-back task alone differentiated verbal memory performance on immediate and delayed word-list recall tasks (Hong Kong List Learning Test). The authors found that the low-activation group performed worse on both immediate and delayed verbal recall trials.

Three studies included a dual-task paradigm [40,41,42]. Some authors quantified peak HbO (highest value) and HbR (lowest value) concentrations in the left prefrontal cortex during simultaneous motor (walking) and cognitive (counting backward) tasks in cognitively healthy, aSCD, and dementia groups [42]. The aSCD group exhibited higher HbO concentrations and lower HbR concentrations than the cognitively healthy and dementia groups during the dual task. Likewise, another study measured HbO and HbR concentrations in the prefrontal cortex of cognitively healthy and SCD participants under two experimental conditions [40]. In Experiment 1, which combined walking and an n-back task, no significant group differences in hemodynamic activity were observed. In Experiment 2, involving finger tapping and an n-back task, individuals with SCD exhibited higher HbO concentrations than cognitively healthy participants during both motor and dual-task conditions. Furthermore, both groups showed increased HbR concentrations and longer reaction times during the dual-task condition compared with the single motor task. Subsequently, other authors assessed HbO and HbR concentrations in the prefrontal cortex of cognitively healthy and SCD groups under different dual-task conditions [41]. During the combination of an n-back task and finger tapping, the entire sample showed higher HbO and HbR concentrations than during the single cognitive task. In the second condition, which combined finger tapping and a double-digit visual sequence task, individuals with SCD exhibited higher HbO and HbR concentrations than cognitively healthy participants. In both groups, dual-task performance was associated with longer reaction times, reduced visual accuracy, greater response variability, and lower motor accuracy.

Finally, two studies investigated prefrontal oxygenation during a semantic verbal fluency task [43,45]. One of them measured HbO concentrations in individuals with aSCD, MCI, and Alzheimer’s disease [45]. The aSCD group showed greater HbO concentrations in the bilateral dorsolateral prefrontal cortex than the MCI and Alzheimer’s disease groups. Likewise, another study assessed HbO concentrations in the prefrontal cortex of individuals with SCD and MCI [43]. Participants with SCD exhibited higher HbO concentrations during the initial phase of the task. Additionally, they showed smaller variations in hemodynamic activity throughout the assessment than those with MCI.

Overall, these findings suggest that individuals with SCD showed higher HbO concentrations during semantic verbal fluency tasks and dual-task paradigms than cognitively healthy individuals and those with objective cognitive impairment.

#### 3.2.4. Language

Only one study incorporated a language task [38]. These authors investigated FC using HbO and HbR time-series data in cognitively healthy, SCD, and MCI groups during the Boston Naming Test. Both the SCD and cognitively healthy groups showed greater HbO-based FC between the left medial prefrontal cortex and the right orbitofrontal cortex than the MCI group. Similarly, the cognitively healthy and SCD groups exhibited stronger HbR-based FC between the left dorsolateral prefrontal cortex and the left orbitofrontal cortex compared with individuals with MCI. Finally, the SCD group showed poorer cognitive performance than the cognitively healthy group, but they achieved higher naming scores and shorter response latencies than the MCI group.

Therefore, cognitively healthy and SCD participants showed greater prefrontal FC than those with MCI during a naming task.

## 4. Discussion

This systematic review aimed to investigate fNIRS-derived hemodynamic correlates associated with SCD. The research findings are discussed in relation to the nine samples that were identified across ten studies. Quality assessment indicated that three studies (targeting two study samples) presented a low risk of bias [37,38,46], four a moderate risk [39,43,44,45], and three a high risk [40,41,42]. Remarkably, several study limitations should be highlighted. First, regarding the study design, all included studies were cross-sectional. This restricts causal inferences regarding the temporal evolution of hemodynamic responses in SCD. Furthermore, 33% of the studies lacked a cognitively healthy control group [43,44,45], which complicates isolating SCD hemodynamic patterns from normal aging, and 55% had small or imbalanced sample sizes [39,40,41,42,43], limiting statistical power and generalizability. Second, diagnostic criteria for SCD lacked standardization. Only 33% of the studies incorporated formal international consensus frameworks [37,38,43,46]. Third, fNIRS protocols presented technical gaps. For instance, 66% of studies did not specify anatomical prefrontal regions [39,40,41,42,43], and 11% omitted fNIRS recordings during cognitive tasks [45]. Fourth, inferential statistical analysis did not consistently adjust for baseline differences (e.g., age and sex), which may compromise the internal validity of group comparisons as both factors independently alter prefrontal oxygenation patterns. Nevertheless, some studies showed notable strengths. These include comprehensive neuropsychological characterization of participants [41] and correlation of fNIRS results with other neuroimaging techniques [45] and different neuropsychological tests [39,44,45,46].

The first aim of this review was to identify patterns of brain activation during the resting-state. In this context, the included studies reported increased FC and higher HbO concentrations in frontal regions in the SCD group compared to cognitively healthy controls [37,43]. This result aligns with recent evidence indicating extensive hyperconnectivity in regions associated with the DMN among individuals with SCD [25]. Conversely, one study found a reduced FC and hemodynamic activity in prefrontal regions in subjects with SCD compared to cognitively healthy controls [46]. A similar functional pattern of dorsolateral prefrontal cortex has been reported in subjects with early-onset Alzheimer’s disease during the resting-state [47]. Taken together, these findings suggest that SCD may be characterized by increased regional HbO levels alongside heterogeneous FC.

The second aim was to assess hemodynamic activity during cognitive performance. In a global cognition task, lower prefrontal FC was observed in the SCD group compared to cognitively healthy controls [38]. Moreover, individuals with SCD showed an intermediate cognitive performance between cognitively healthy and MCI groups. Regarding the memory domain, one study observed a severity-dependent response where both mild aSCD and cognitively healthy groups showed higher HbO concentrations in the prefrontal cortex compared to the severe aSCD group [39]. Similarly, another study found a lower FC in the prefrontal cortex in individuals with SCD compared to the cognitively healthy group [38]. These results may align with fMRI studies reporting both greater task-related activity in the dorsolateral prefrontal cortex and lower activity in the superior parietal and hippocampus in individuals with SCD during encoding and retrieval tasks [48]. Memory performance also varied across groups, with SCD individuals exhibiting either an intermediate cognitive performance between cognitively healthy and MCI groups [38] or no significant differences compared to a cognitively healthy group [39]. Another study found no hemodynamic or neuropsychological differences between SCD and MCI groups, which was attributed to the low cognitive demands of the task employed [43]. Notably, the latter study did not include a cognitively healthy group. In the executive function domain, during dual-tasking, the aSCD group showed greater HbO versus reduced HbR concentrations in the prefrontal cortex compared to the cognitively healthy controls [42]. In contrast, other studies observed concurrent increases in both HbO and HbR concentrations in SCD compared to cognitively healthy controls [40,41]. These conflicting HbR directions may reflect differences in cerebral blood flow or variations in task demands [40,41,49]. In semantic verbal fluency tasks, the SCD group showed higher HbO concentrations in the prefrontal cortex than groups with objective cognitive impairment [43,45], but these studies lacked cognitively healthy groups and did not assess task performance during fNIRS recording [43,45]. Finally, higher n-back accuracy in aSCD was associated with higher prefrontal HbO concentrations [44]. Regarding language processing, both SCD and cognitively healthy groups exhibited greater prefrontal FC than the MCI group. Nevertheless, the SCD group showed lower neuropsychological performance compared to the cognitively healthy group [38].

The available evidence suggests distinct prefrontal hemodynamic patterns across healthy aging, SCD, and objective cognitive impairment. However, current literature remains limited and methodologically inconsistent. Many studies lacked data on neuropsychological performance and cognitively healthy control groups. Therefore, establishing a specific hemodynamic pattern for SCD remains challenging.

Two plausible hypotheses have been considered to explain prefrontal functioning in individuals with SCD during resting-state and cognitive task performance. On the one hand, some authors suggest that individuals with SCD recruit additional prefrontal hemodynamic resources [39,40,41,42,43]. This heightened baseline activity may serve as a functional buffer, preparing the brain to meet subsequent cognitive demands and thereby supporting task performance. This aligns with the Scaffolding Theory of Aging and Cognition (STAC) [50], the Compensation-Related Utilization of Neural Circuits Hypothesis (CRUNCH) [51], and the Hemispheric Asymmetry Reduction in Older Adults (HAROLD) model [52]. Accordingly, fMRI studies have reported prefrontal hyperactivation in response to medial temporal lobe inefficiency during verbal, spatial, working memory, and word-retrieval tasks in healthy older adults [52,53]. While individuals with mild SCD may show increased prefrontal recruitment, those with higher complaint severity may show reduced activation and connectivity under higher cognitive demands [39,40]. This pattern would mimic that observed in individuals with objective cognitive impairment [40,47]. However, testing these hypotheses requires future research to address key methodological points. Future studies should (i) adopt longitudinal and well-powered designs to track fNIRS trajectories in SCD; (ii) improve SCD operationalization to ensure diagnostic consistency; (iii) examine the impact of symptom severity on brain function; (iv) consistently include healthy control groups to establish a baseline for brain activity; (v) include representative samples and balance group sample sizes; (vi) implement challenging cognitive tasks during fNIRS recording to better detect group differences; and (vii) report hemodynamic responses alongside task performance scores.

This systematic review is not exempt from limitations. First, articles in languages other than English or Spanish were excluded, as most of the scientific literature is published in English, and the authors’ language proficiency was the basis for the study selection. Second, study search and identification were conducted by a single author; however, study eligibility was discussed among several co-authors. Third, limited literature and methodological heterogeneity complicate drawing definitive conclusions regarding fNIRS neural patterns in SCD. Therefore, this original review serves as an initial overview of this emerging research area, and the results should be interpreted with caution.

## 5. Conclusions

Based on the evidence reviewed, we conclude that fNIRS studies of SCD remain scarce and methodologically heterogeneous, making it difficult to establish a specific hemodynamic pattern for SCD. Moreover, SCD exhibits heterogeneous hemodynamic responses under resting-state and cognitive task conditions. The severity of subjective complaints and task difficulty may serve as useful moderators. Although the scientific evidence in this field remains limited, these observations provide a valuable agenda for future research. Ultimately, understanding early hemodynamic patterns in SCD may help identify individuals at higher risk for cognitive impairment/dementia.

## Figures and Tables

**Table 1:** Main characteristics and key findings of the studies.

Authors and Country	JBI Overall Risk of Bias	Sample	Criteria for SCD	Experimental Condition	Covariates ^a^	Hemodynamic Measure	ROI	Hemodynamic Results	Between-Group Comparisons ^b^
Lee et al. 2024 [39] (China)	Moderate	37 CH 24 mild aSCD 33 moderate aSCD 29 severe aSCD 15 aMCI 13 naMCI	AMIC = 3 (mild aSCD) AMIC = 4 (moderate aSCDm) AMIC = 5 (severe aSCD)	Visual memory span	Age, sex, education level	HbO	Prefrontal cortex	HbO (whole task): severe aSCD < CH and mild aSCD HbO when difficulty increase: moderate aSCD > severe aSCD	*d* = 0.70, *p* = 0.005; *d* = 0.97, *p* = 0.001 *d* * = [0.51, 0.61], p * = [0.021, 0.049]
Pu, Ni et al. 2024 [37] (China)	Low	55 CH 80 SCD 111 MCI	SCD-I (2014), SCD-Q9 ≥ 5	Resting-state	Matched by design (age, sex)	FC (HbO, HbR, HbT)	DLPFC, OFC	FC (HbR) between left DLPFC and right OFC: SCD > CH	LSD-t = 3.61, *p* < 0.001
Pu, Fang et al. 2025 [38] (China)	Low	55 CH 80 SCD 111 MCI	SCD-I (2014), SCD-Q9 ≥ 5	Cognitive testing	Matched by design (age, sex)	FC (HbO, HbR, HbT)	DLPFC, OFC, VLPFC MPFC	MMSE: FC (HbO) between right DLPFC and right OFC: CH > SCD FC (HbR) between left DLPFC and left VLPFC: CH > SCD FC (HbT) between right DLPFC and left OFC: CH > SCD HVLT: FC (HbR) between left DLPFC and right MPFC: CH > SCD BNT FC (HbO) between left MPFC and right OFC: MCI < SCD FC (HbR) between left DLPFC and left OFC: MCI < SCD	MMSE: LSD-t = 2.89, *p* = 0.004 LSD-t = 2.05, *p* = 0.041 LSD-t = 2.46, *p* = 0.015 HVLT: LSD-t = 3.29, *p* = 0.001 BNT: LSD-t = 2.72, *p* = 0.007 LSD-t = 2.41, *p* = 0.017
Salzman et al. 2023 [40] (Canada)	High	10 CH 10 SCD	Concern about memory/cognitive problems MoCA ≥ 26	Condition 1: Single (n-back, walking) and Dual (n-back + walking) tasks Condition 2: Single (n-back, finger tapping) and Dual (n-back + finger tapping) tasks	Matched by design (age, education level, MoCA, cardiovascular disease, physical activity, depression)	HbO, HbR	Prefrontal cortex	Condition 1: no significant ≠ between groups Condition 2: HbO in finger tapping during dual task: SCD > CH	Condition 2: *M* = 0.074 ± 0.016, *M* = 0.002 ± 0.014 (*p* = 0.002)
Salzman et al. 2026 [41] (Canada)	High	18 CH 24 SCD	Concern about memory/cognitive problems MoCA ≥ 26	Condition 1: Single (finger tapping, n-back) and Dual (n-back + finger tapping) tasks Condition 2: Single (finger tapping, visual task) and Dual (finger tapping + visual task) tasks	Anxiety, depression	HbO, HbR	Prefrontal cortex	Condition 1: no significant ≠ between groups Condition 2: HbO and HbR in dual task: SCD > CH	Condition 2: HbO: *F* (1,40) = 4.38, *p* = 0.04, η^2^ = 0.099. Post hoc: *p* = 0.016 HbR: *F* (1,40) = 5.49, *p* = 0.001, η^2^ = 0.025 ^c^. Post hoc: *p* = 0.011
Teo et al. 2020 [42] (Australia)	High	26 CH 23 aSCD 9 dementia	Physically healthy Perceived memory worsening MoCA > 24	Single task: walking Dual task: counting backward + walking	Matched by design (education level, height, weight)	HbO, HbR	Left prefrontal cortex	Single task: no significant ≠ between CH and aSCD Dual task: HbO: aSCD > CH HbR: aSCD < CH and dementia	Dual task: HbO: *M* = 0.90 ± 0.40, *M* = 0.41 ± 0.15 (*p* < 0.001) HbR: *M* = −0.08 ± 0.04, *M* = −0.02 ± 0.01 (*p* < 0.001)
Wang et al. 2024 [43] (China)	Moderate	13 SCD 20 MCI	SCD-I (2020)	Resting-state, SVFT, MemTrax	Matched by design (age, sex, education level)	FC (HbO), HbO	Prefrontal cortex	Resting-state: FC (HbO): SCD > MCI SVFT: HbO: SCD > MCI	Resting-state: *t* = 2.09–3.16, *p* < 0.05 SVFT: *t* (31) = 3.29, *p* = 0.02
Yeung et al. 2022 [44] (China)	Moderate	12 aSCD high bilateral activation 24 aSCD low bilateral activation	Perceived memory problems, ≥3 short memory questionnaires for the Chinese <1.5 SD, HKLLT	n-back	Matched by design (age, sex, education level, daily functioning)	HbO	Prefrontal cortex	2-back: HbO: aSCD high bilateral activation > aSCD low bilateral activation	*F* (1, 34) = 4.57, *p* = 0.040, η^2^*p* = 0.12. Post hoc: ts > 5.74, *p*s < 0.001
Yoon et al. 2023 [45] (South Korea)	Moderate	24 aSCD 30 aMCI 29 naMCI 24 AD	Consecutive referrals to a psychiatry clinic due to suspicion of cognitive impairment	SVFT	Age	HbO	DLPFC	HbO in left and right DLPFC: aSCD > aMCI, naMCI, and AD.	*F* (3, 98) = 3.01, *p* = 0.034. Post hoc: *p* = 0.061, *p* = 0.032, *p* = 0.027
Zhang et al. 2024 [46] (China)	Low	78 CH 123 SCD 142 MCI 52 AD	SCD-I (2014)	Resting-state	Age, sex, education level	HbO FC	Bilateral FPC, DLPFC, OFC, VLPFC	HbO in right and left FPC cortex: SCD > CH HbO in left DLPFC: SCD > CH Intrahemispheric FC: CH > SCD Interhemispheric FC: CH > SCD	*M* right FPC: −0.33 ± 1.15, 0.09 ± 0.67 (*p* < 0.05); *M* left FPC: −0.51 ± 1.30, −0.02 ± 0.72 (*p* < 0.05) *M* left DLPFC: −0.51 ± 1.32, −0.09 ± 0.61 (*p* < 0.05) Intrahemispheric: B * = [−0.274, −0.152], *t* * = [−4.083, −2.400], *p* * = [<0.001, 0.017]; but left FPC–left VLPFC: B = 0.129, *t* = 2.052, *p* = 0.041 Interhemispheric: B * = [−0.241, −0.137], *t* * = [−3.442, −2.057], *p* * = [.001, 0.04]; but left VLPFC–right FPC: B = 0.177, *t* = 2.976, *p* = 0.003

Note: ^a^ Variables included in inferential statistical models for adjustment or controlled via a priori matching. ^b^ All reported statistics reflect those applied in the original papers to evaluate group differences. The main objective was to report effect sizes (e.g., Cohen’s *d*; partial eta-squared [η^2^_p_]; and eta-squared [η^2^]). When effect sizes were unavailable, we reported descriptive statistics (e.g., means [*M*]), inferential test statistics (e.g., ANOVA F-values [*F*], t-statistics [*t*], and Fisher’s Least Significant Difference post hoc contrasts [LSD-t]), or regression coefficients [*B*]. ^c^ Values for Salzman et al. are reported as printed in the original source. Note that the reported *p*-value is not mathematically consistent with the reported *F*-statistic, suggesting an internal error in the original publication. AD (Alzheimer’s disease), aMCI (amnestic MCI), AMIC (Abbreviated Memory Inventory for Chinese), aSCD (amnestic subjective cognitive decline), BNT (Boston Naming Test), CDR (Clinical Dementia Rating), DLPFC (dorsolateral prefrontal cortex), FC (functional connectivity), FPC (frontopolar cortex), HbO (oxygenated hemoglobin), HbR (deoxygenated hemoglobin), HbT (total hemoglobin), CH (cognitively healthy), HKLLT (Hong Kong List Learning Test), HVLT (Hopkins Verbal Learning Test), MCI (mild cognitive impairment), MMSE (Mini-Mental State Examination), MPFC (medial prefrontal cortex), MoCA (Montreal Cognitive Assessment), naMCI (non-amnestic MCI), OFC (orbitofrontal cortex), SCD (subjective cognitive decline), SCD-I (Subjective Cognitive Decline Initiative), SCD-Q9 (Subjective Cognitive Decline Questionnaire 9), SD (standard deviation), SVFT (Semantic Verbal Fluency Test), and VLPFC (ventrolateral prefrontal cortex). * These values are reported in ranges to improve readability.

## Data Availability

No new data were created or analyzed in this study. Data sharing is not applicable to this article.

## Supplementary Materials

The following supporting information can be downloaded at: https://www.mdpi.com/article/10.3390/brainsci16101130/s1, Table S1. Quality assessment by the JBI Critical Appraisal Checklist for Analytical Cross-Sectional Studies. Table S2. Cognitive performance results of the included studies. Table S3. PRISMA checklist. Table S4. PRISMA-S checklist.

## Abbreviations

The following abbreviations are used in this manuscript:

aSCD	Amnestic subjective cognitive decline
CRUNCH	Compensation-related utilization of neural circuits
DMN	Default mode network
FC	Functional connectivity
fMRI	Functional magnetic resonance imaging
fNIRS	Functional near-infrared spectroscopy
HAROLD	Hemispheric asymmetry reduction in older adults
HbO	Oxyhemoglobin
HbR	Deoxyhemoglobin
MCI	Mild cognitive impairment
MMSE	Mini-Mental State Examination
MoCA	Montreal Cognitive Assessment
MRI	Magnetic resonance imaging
PET	Positron emission tomography
SCD	Subjective cognitive decline
SCD-I	Subjective Cognitive Decline Initiative
SPECT	Single-photon emission computed tomography
STAC	Scaffolding Theory of Aging and Cognition
