# Supplementary material for: Cerebral Hemodynamic Patterns in People with Subjective Cognitive Decline: A Systematic Review Based on Functional Near-Infrared Spectroscopy
Source: Brain Sci. 2026 Oct 20;16(10):1130. doi: 10.3390/brainsci16101130 (PMC13702552; doi:10.3390/brainsci16101130)
Supplement: Supplementary file 1 [file brainsci-16-01130-s001.zip › brainsci-4505972-supplementary.pdf]

Supplementary Materials

Table S1. Quality assessment by the JBI Critical Appraisal Checklist for Analytical Cross-Sectional Studies

Authors	Q1: Inclusion criteria	Q2: Condition criteria	Q3: Exposure measurement	Q4: Outcome validity	Q5: Confounding identification	Q6: Confounding control	Q7: Statistical appropriateness	Q8: Study setting
**Lee et al. 2024** [39]	Yes	Yes	Yes	Unclear	Yes	Yes	Unclear	Yes
**Pu, Ni et al. 2025** [37]	Yes	Yes	Yes	Yes	Yes	Yes	Yes	Yes
**Pu, Fang et al. 2025** [38]	Yes	Yes	Yes	Yes	Yes	Yes	Yes	Yes
**Salzman et al. 2023** [40]	Yes	Unclear	Yes	Unclear	Yes	No	Unclear	Yes
**Salzman et al. 2026** [41]	Yes	Unclear	Yes	Unclear	Yes	Yes	Unclear	Yes
**Teo et al. 2020** [42]	Yes	Unclear	Yes	Unclear	Yes	No	Unclear	Yes
**Wang et al. 2024** [43]	Yes	Yes	Yes	Unclear	Yes	Yes	Uncelar	Yes
**Yeung et al. 2022** [44]	Yes	Unclear	Yes	Unclear	Yes	Yes	Yes	Yes
**Yoon et al. 2023** [45]	Yes	Unclear	Yes	Unclear	Yes	Yes	Yes	Yes
**Zhang et al. 2024** [46]	Yes	Yes	Yes	Yes	Yes	Yes	Yes	Yes

Note: Q1 (Were the criteria for inclusion in the sample clearly defined?), Q2 (Were objective, standard criteria used for measurement of the condition?), Q3 (Was the exposure measured in a valid and reliable way?), Q4 (Were the outcomes measured in a valid and reliable way?), Q5 (Were confounding factors identified?), Q6 (Were strategies to deal with confounding factors stated?), Q7 (Was appropriate statistical analysis used?), Q8 (Were the study subjects and the setting described in detail?).

Table S2. Cognitive performance results of the included studies

Authors and country	Cognitive measures	Behavioral results
Lee et al. 2024 [39]	Visual memory span	No group differences among aSCD mild, aSCDm, aSCDs and CH.
Pu, Ni et al. 2025 [37]	MMSE, TMT-A, TMT-B, BNT, HVLT, CDT	MCI < CH and SCD.
Pu, Fang et al. 2025 [38]	MMSE, HVLT, BNT	MMSE: CH and SCD > MCI<br>Response time: CH < SCD and MCI; SCD < MCI<br>HVLT: SCD > MCI; CH > SCD and MCI<br>BNT: SCD > MCI; CH > SCD and MCI<br>Response time: CH < SCD and MCI; SCD < MCI
Salzman et al. 2023 [40]	Condition 1: Single (n-back, walking) and Dual (n-back + walking) tasks<br>Condition 2: Single (n-back, finger tapping) and Dual (n-back + finger tapping) tasks	Condition 1:<br>Gait speed in CH: Dual task < single task<br>Condition 2:<br>n-back response time in CH and SCD: dual task > single task
Salzman et al. 2026 [41]	Condition 1: Single (finger tapping, n-back) and Dual (n-back + finger tapping) tasks<br>Condition 2: Single (finger tapping, visual task) and Dual (finger tapping + visual task) tasks	Both conditions:<br>n-back response time in CH and SCD: single task < dual task<br>Condition 1:<br>Tapping variability in CH and SCD: single task < dual task<br>Tapping accuracy in SCD and CH: single task > dual task<br>Condition 2:<br>Visual task accuracy in CH y SCD: single task > dual task.<br>Tapping accuracy in SCD: single task > dual task
Teo et al. 2020 [42]	Single task (walking)<br>Dual task (counting backwards + walking)	Step lenght and velocity in CH y aSCD: Dual task < Single task<br>Total number of counting responses in dual task: dementia < CH y aSCD
Wang et al. 2024 [43]	MemTrax	No significant differences between groups
Yeung et al. 2022 [44]	n-back	n-back accuracy in aSCD: 0-back > 2-back<br>0-back and 2-back accuracy: high bilateral activation > low bilateral activation<br>n-back response time in aSCD: 2-back > 0-back.

Yoon et al. 2023 [45]	SVFT	Not recorded.
Zhang et al. 2024 [46]	MMSE, MoCA	SCD > MCI; MCI > AD

Note: AD (Alzheimer´s disease), aMCI (amnestic MCI), aSCD (amnestic subjective cognitive decline), aSCDm and aSCDs (moderate/severe amnestic subjective cognitive decline), BNT (Boston Naming Test), CDT (Clock Drawing Test), CH (cognitively healthy), HVLT (Hopkins Verbal Learning Test), MCI (mild cognitive impairment), MMSE (Mini-Mental State Examination), MoCA (Montreal Cognitive Assessment), SCD (subjective cognitive decline), SVFT (Semantic Verbal Fluency Test), TMT-A/TMT-B (Trail Making Test A/B), VFT (Verbal Fluency Test).

Table S3. PRISMA Checklist

Section and Topic	Item #	Checklist item	Location where item is reported
**TITLE**			
Title	1	Identify the report as a systematic review.	p. 1
**ABSTRACT**			
Abstract	2	See the PRISMA 2020 for Abstracts checklist.	p. 1
**INTRODUCTION**			
Rationale	3	Describe the rationale for the review in the context of existing knowledge.	pp. 2-3
Objectives	4	Provide an explicit statement of the objective(s) or question(s) the review addresses.	p. 3
**METHODS**			
Eligibility criteria	5	Specify the inclusion and exclusion criteria for the review and how studies were grouped for the syntheses.	p. 3
Information sources	6	Specify all databases, registers, websites, organisations, reference lists and other sources searched or consulted to identify studies. Specify the date when each source was last searched or consulted.	pp. 3-4
Search strategy	7	Present the full search strategies for all databases, registers and websites, including any filters and limits used.	p. 4
Selection process	8	Specify the methods used to decide whether a study met the inclusion criteria of the review, including how many reviewers screened each record and each report retrieved, whether they worked independently, and if applicable, details of automation tools used in the process.	p. 4
Data collection process	9	Specify the methods used to collect data from reports, including how many reviewers collected data from each report, whether they worked independently, any processes for obtaining or confirming data from study investigators, and if applicable, details of automation tools used in the process.	p. 4
Data items	10a	List and define all outcomes for which data were sought. Specify whether all results that were compatible with each outcome domain in each study were sought (e.g. for all measures, time points, analyses), and if not, the methods used to decide which results to collect.	p. 4
	10b	List and define all other variables for which data were sought (e.g. participant and intervention characteristics, funding sources). Describe any assumptions made about any missing or unclear information.	p. 4
Study risk of bias assessment	11	Specify the methods used to assess risk of bias in the included studies, including details of the tool(s) used, how many reviewers assessed each study and whether they worked independently, and if applicable, details of automation tools used in the process.	p. 4
Effect measures	12	Specify for each outcome the effect measure(s) (e.g. risk ratio, mean difference) used in the synthesis or presentation of results.	p. 4
Synthesis methods	13a	Describe the processes used to decide which studies were eligible for each synthesis (e.g. tabulating the study intervention characteristics and comparing against the planned groups for each synthesis (item #5)).	p. 4
	13b	Describe any methods required to prepare the data for presentation or synthesis, such as handling of missing summary statistics, or data conversions.	NA
	13c	Describe any methods used to tabulate or visually display results of individual studies and syntheses.	p. 4
	13d	Describe any methods used to synthesize results and provide a rationale for the choice(s). If meta-analysis was performed, describe the model(s), method(s) to identify the presence and extent of statistical heterogeneity, and software package(s) used.	NA
	13e	Describe any methods used to explore possible causes of heterogeneity among study results (e.g. subgroup analysis, meta-regression).	NA
	13f	Describe any sensitivity analyses conducted to assess robustness of the synthesized results.	NA
Reporting bias assessment	14	Describe any methods used to assess risk of bias due to missing results in a synthesis (arising from reporting biases).	NA

Section and Topic	Item #	Checklist item	Location where item is reported
Certainty assessment	15	Describe any methods used to assess certainty (or confidence) in the body of evidence for an outcome.	p. 4
**RESULTS**			
Study selection	16a	Describe the results of the search and selection process, from the number of records identified in the search to the number of studies included in the review, ideally using a flow diagram.	p. 5
	16b	Cite studies that might appear to meet the inclusion criteria, but which were excluded, and explain why they were excluded.	p. 5
Study characteristics	17	Cite each included study and present its characteristics.	p. 5
Risk of bias in studies	18	Present assessments of risk of bias for each included study.	p. 5
Results of individual studies	19	For all outcomes, present, for each study: (a) summary statistics for each group (where appropriate) and (b) an effect estimate and its precision (e.g. confidence/credible interval), ideally using structured tables or plots.	p. 5
Results of syntheses	20a	For each synthesis, briefly summarise the characteristics and risk of bias among contributing studies.	p. 5
	20b	Present results of all statistical syntheses conducted. If meta-analysis was done, present for each the summary estimate and its precision (e.g. confidence/credible interval) and measures of statistical heterogeneity. If comparing groups, describe the direction of the effect.	pp. 6-9
	20c	Present results of all investigations of possible causes of heterogeneity among study results.	pp. 10-12
	20d	Present results of all sensitivity analyses conducted to assess the robustness of the synthesized results.	NA
Reporting biases	21	Present assessments of risk of bias due to missing results (arising from reporting biases) for each synthesis assessed.	NA
Certainty of evidence	22	Present assessments of certainty (or confidence) in the body of evidence for each outcome assessed.	NA
**DISCUSSION**			
Discussion	23a	Provide a general interpretation of the results in the context of other evidence.	pp. 12-14
	23b	Discuss any limitations of the evidence included in the review.	p. 12
	23c	Discuss any limitations of the review processes used.	p. 14
	23d	Discuss implications of the results for practice, policy, and future research.	pp. 14
**OTHER INFORMATION**			
Registration and protocol	24a	Provide registration information for the review, including register name and registration number, or state that the review was not registered.	p. 3
	24b	Indicate where the review protocol can be accessed, or state that a protocol was not prepared.	p. 3
	24c	Describe and explain any amendments to information provided at registration or in the protocol.	NA
Support	25	Describe sources of financial or non-financial support for the review, and the role of the funders or sponsors in the review.	p. 14
Competing interests	26	Declare any competing interests of review authors.	p. 14
Availability of data, code and other materials	27	Report which of the following are publicly available and where they can be found: template data collection forms; data extracted from included studies; data used for all analyses; analytic code; any other materials used in the review.	NA

From: Page MJ, McKenzie JE, Bossuyt PM, Boutron I, Hoffmann TC, Mulrow CD, et al. The PRISMA 2020 statement: an updated guideline for reporting systematic reviews. BMJ 2021;372:n71. doi: 10.1136/bmj.n71. This work is licensed under CC BY 4.0. To view a copy of this license, visit https://creativecommons.org/licenses/by/4.0/

Table S4. PRISMA-S Checklist

Section/topic	#	Checklist item	Location(s) Reported
**INFORMATION SOURCES AND METHODS**			
Database name	1	Name each individual database searched, stating the platform for each.	p. 3
Multi-database searching	2	If databases were searched simultaneously on a single platform, state the name of the platform, listing all of the databases searched.	NA
Study registries	3	List any study registries searched.	NA
Online resources and browsing	4	Describe any online or print source purposefully searched or browsed (e.g., tables of contents, print conference proceedings, web sites), and how this was done.	NA
Citation searching	5	Indicate whether cited references or citing references were examined, and describe any methods used for locating cited/citing references (e.g., browsing reference lists, using a citation index, setting up email alerts for references citing included studies).	p. 4
Contacts	6	Indicate whether additional studies or data were sought by contacting authors, experts, manufacturers, or others.	p. 4
Other methods	7	Describe any additional information sources or search methods used.	NA
**SEARCH STRATEGIES**			
Full search strategies	8	Include the search strategies for each database and information source, copied and pasted exactly as run.	p. 4
Limits and restrictions	9	Specify that no limits were used, or describe any limits or restrictions applied to a search (e.g., date or time period, language, study design) and provide justification for their use.	p. 4
Search filters	10	Indicate whether published search filters were used (as originally designed or modified), and if so, cite the filter(s) used.	p. 4
Prior work	11	Indicate when search strategies from other literature reviews were adapted or reused for a substantive part or all of the search, citing the previous review(s).	NA
Updates	12	Report the methods used to update the search(es) (e.g., rerunning searches, email alerts).	NA
Dates of searches	13	For each search strategy, provide the date when the last search occurred.	p. 4
**PEER REVIEW**			
Peer review	14	Describe any search peer review process.	p. 3
**MANAGING RECORDS**			
Total Records	15	Document the total number of records identified from each database and other information sources.	p. 5
Deduplication	16	Describe the processes and any software used to deduplicate records from multiple database searches and other information sources.	p. 4

PRISMA-S: An Extension to the PRISMA Statement for Reporting Literature Searches in Systematic Reviews.

Rethlefsen ML, Kirtley S, Waffenschmidt S, Ayala AP, Moher D, Page MJ, Koffel JB, PRISMA-S Group. Last updated February 27, 2020.
